# Supplementary material for: Maximizing the clinical utility and performance of cytology samples for comprehensive genetic profiling
Source: Nat Commun. 2025 Jan 2;16:116. doi: 10.1038/s41467-024-55456-8 (PMC11696557; doi:10.1038/s41467-024-55456-8)
Supplement: Supplementary file 1 — Supplementary Information [file 41467_2024_55456_MOESM1_ESM.pdf]

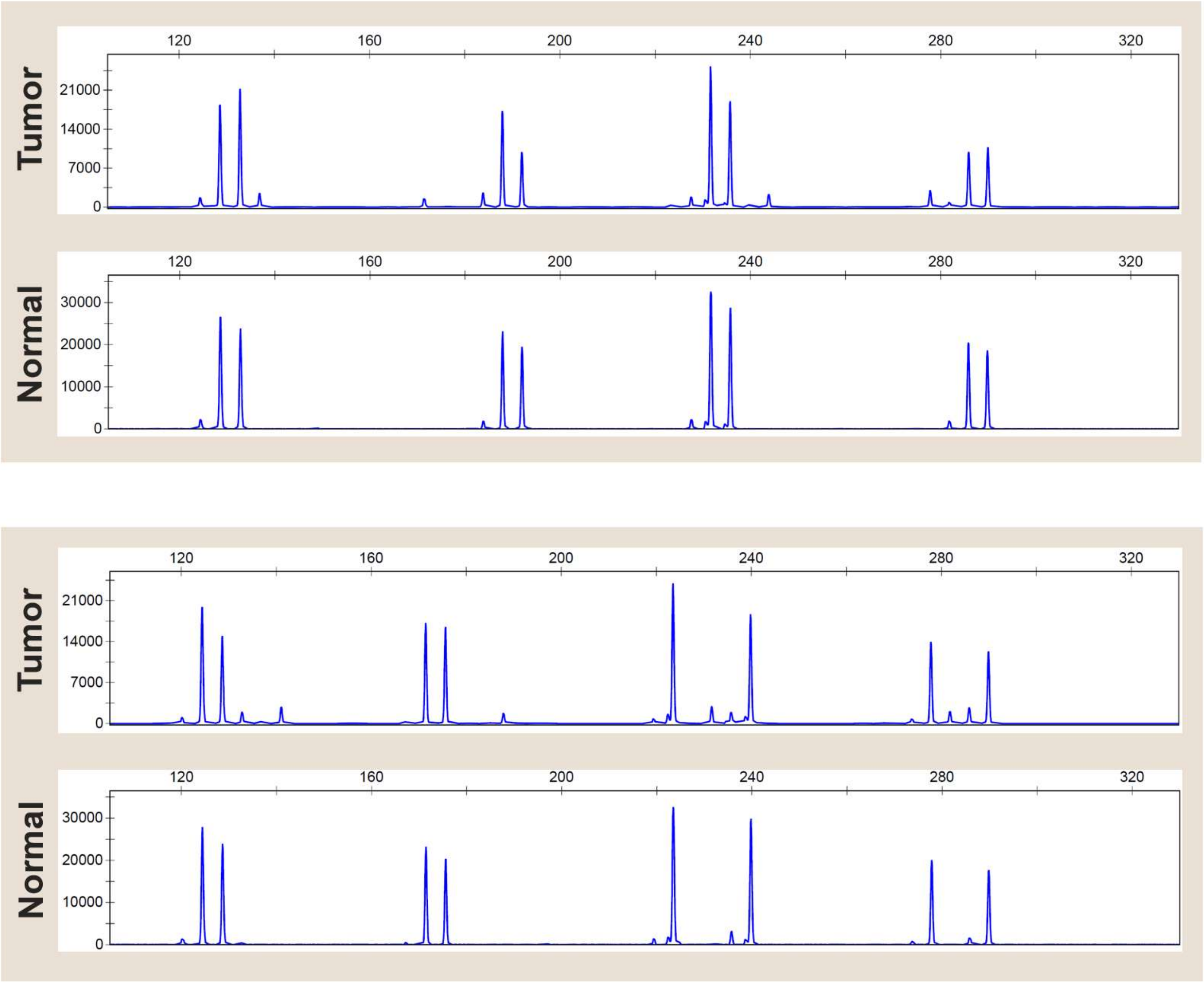

Supplementary Figure 1: Representative short tandem repeat (STR) analysis of CB samples with matched plasma normal samples demonstrating low- and high-level contamination by non-patient DNA as evidenced by additional peaks in the tumor sample not seen in the normal sample.

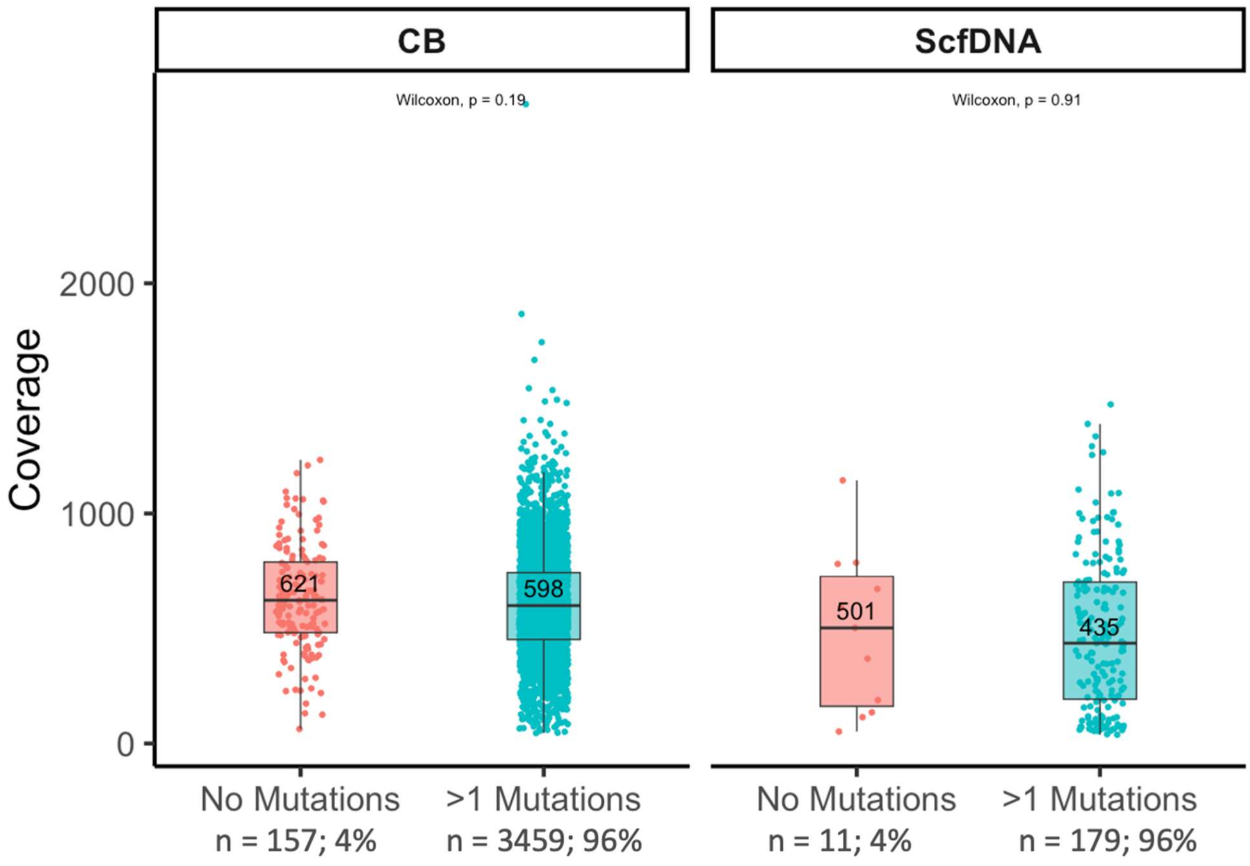

Supplementary Figure 2: The distribution of total sample coverage between cytology cases that identified no somatic alterations and those that identified at least one somatic alteration compared for CB (p = 0.19) and ScfDNA (*p* = 0.91) samples. The *p value* was assessed by a two-tailed Mann-Whitney test. All boxplots show the median (center line with value) and 25th and 75th percentiles (bounding box) along with the 1.5 interquartile range (whiskers).

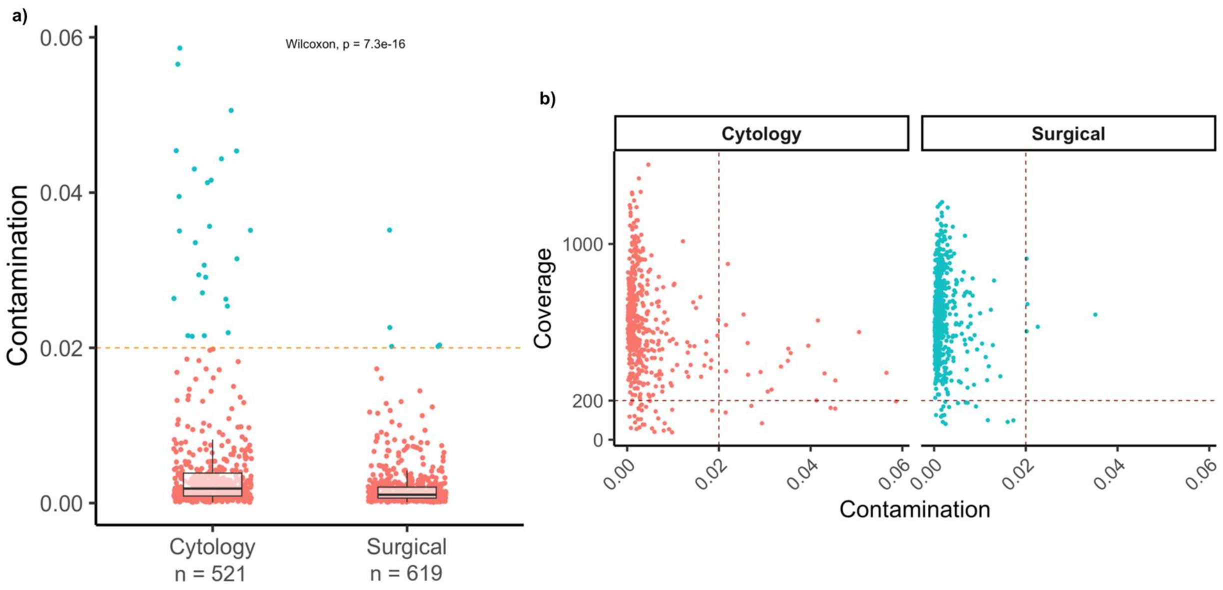

Supplementary Figure 3: a Non-patient DNA contamination rate distribution of cytology samples and any matching surgical sample from the same patient tumor. The horizontal dashed line indicates a contamination rate of 2% for which samples above this threshold has a significant concern for contamination. While the majority of samples for both cytology and paired surgical samples were below 2%, there was a higher total number of samples above this threshold for cytology samples (26 cytology samples vs 5 surgical samples). The *p value* was assessed by a two-tailed Mann-Whitney test. All boxplots show the median (center line with value) and 25th and 75th percentiles (bounding box) along with the 1.5 interquartile range (whiskers). **b** The distribution of contamination rates between cytology sample and their matching surgical sample charted by sample coverage. The dashed vertical line indicates the threshold contamination rate of 0.02 and the dashed horizontal line denotes the threshold for adequate coverage (200x). Samples in the top-right portion indicate a high contamination rate in the face of adequate coverage, whereas samples in the bottom-right had low coverage that may falsely elevate the contamination rate.

Supplementary Table 1: Optimization strategies implemented for cytology/small biopsy samples.

Optimization Method	Laboratory	Implementation Year	Benefit Outcome	Description
Modified HistoGel Cell-Block	Cytology	2014	Increased tissue/tumor yield in cell block samples	FFPE cytology sample prepared by a HistoGel cell bock method with the modification of pretreatment by 95% ethanol.
Supernatant cfDNA Protocol	Cytology	2014	Provides an additional source of genomic material utlizing residual CytoLyt supernatant fluid.	Residual CytoLyt supernatant that is traditionally discarded is further spun down to a pellet and treated with isopropanol. Additional genomic material is therefore rescued in cases where all other tissue sources are exhausted.
Mineral Oil Deparaffinization	Molecular	2014	Increased DNA yield in the extraction of FFPE material. Reduces hands-on labor and processing of FFPE samples.	Deparaffinization of FFPE samples carried out with mineral oil.
Bead-Extraction Optimization	Molecular	2018-2019	Improved DNA extraction and purification	Bead-extraction procedures optimized for DNA extraction and purification prior to library preparation.
Dual index Sequencing	Molecular	2020	Increased accuracy of sample identification and improved efficiency in processing.	Two additional reads are included in a sequencing run allowing for greater accuracy and efficiency. With an increase number of unique barcode combinations, sequencing errors are decreased with reduced index hopping and misaligned/misread reads.
Lower DNA Yield Threshold	Molecular	2021	Due to improved extraction and sequencing methods, a lower DNA extraction yield could be tolerated for sequencing in samples that otherwise would have been inadequate.	The minimum DNA extraction yield required to proceed with sequenccing was lowered from 0.9 ng/mL (49 ng) to 0.54 ng/mL (<30 ng).
