# Supplementary material for: Maximizing the clinical utility and performance of cytology samples for comprehensive genetic profiling
Source: Nat Commun. 2025 Jan 2;16:116. doi: 10.1038/s41467-024-55456-8 (PMC11696557; doi:10.1038/s41467-024-55456-8)
Supplement: Supplementary file 3 — Description of Additional Supplementary Files [file 41467_2024_55456_MOESM3_ESM.pdf]

Description of Additional Supplementary Files

Supplementary Data 1 - The number of samples of the profiled tumor types and their sample preparation type in the study cohort.
