# Supplementary material for: Maximizing the clinical utility and performance of cytology samples for comprehensive genetic profiling
Source: Nat Commun. 2025 Jan 2;16:116. doi: 10.1038/s41467-024-55456-8 (PMC11696557; doi:10.1038/s41467-024-55456-8)
Supplement: Supplementary file 5 — Reporting Summary [file 41467_2024_55456_MOESM5_ESM.pdf]

nature portfolio

Corresponding author(s): David Kim

Last updated by author(s): Nov 21, 2024

Reporting Summary

Nature Portfolio wishes to improve the reproducibility of the work that we publish. This form provides structure for consistency and transparency in reporting. For further information on Nature Portfolio policies, see our Editorial Policies and the Editorial Policy Checklist.

Statistics

For all statistical analyses, confirm that the following items are present in the figure legend, table legend, main text, or Methods section.

n/a	Confirmed	
☐	☒	The exact sample size (*n*) for each experimental group/condition, given as a discrete number and unit of measurement
☐	☒	A statement on whether measurements were taken from distinct samples or whether the same sample was measured repeatedly
☐	☒	The statistical test(s) used AND whether they are one- or two-sided<br>*Only common tests should be described solely by name; describe more complex techniques in the Methods section.*
☒	☐	A description of all covariates tested
☒	☐	A description of any assumptions or corrections, such as tests of normality and adjustment for multiple comparisons
☐	☒	A full description of the statistical parameters including central tendency (e.g. means) or other basic estimates (e.g. regression coefficient) AND variation (e.g. standard deviation) or associated estimates of uncertainty (e.g. confidence intervals)
☐	☒	For null hypothesis testing, the test statistic (e.g. *F*, *t*, *r*) with confidence intervals, effect sizes, degrees of freedom and *P* value noted<br>*Give P values as exact values whenever suitable.*
☒	☐	For Bayesian analysis, information on the choice of priors and Markov chain Monte Carlo settings
☒	☐	For hierarchical and complex designs, identification of the appropriate level for tests and full reporting of outcomes
☐	☒	Estimates of effect sizes (e.g. Cohen's *d*, Pearson's *r*), indicating how they were calculated

Our web collection on statistics for biologists contains articles on many of the points above.

Software and code

Policy information about availability of computer code

Data collection	no software was used for data collection.
Data analysis	R version 4.1.1 was used to analyze the data for this study.

For manuscripts utilizing custom algorithms or software that are central to the research but not yet described in published literature, software must be made available to editors and reviewers. We strongly encourage code deposition in a community repository (e.g. GitHub). See the Nature Portfolio guidelines for submitting code & software for further information.

Data

Policy information about availability of data

All manuscripts must include a data availability statement. This statement should provide the following information, where applicable:

- Accession codes, unique identifiers, or web links for publicly available datasets
- A description of any restrictions on data availability
- For clinical datasets or third party data, please ensure that the statement adheres to our policy

The raw sequencing data for MSK-IMPACT analysis is protected and cannot be broadly available due to privacy laws. Patient consent to deposit the raw sequencing data was not obtained. Data supporting the analysis for all samples, including all genomic results, in this study are included with this published Article, Supplementary Information, and Source Data files. Unique sample IDs of the study samples are provided in the Source Data files and available for query on

cBioportal: https://www.cbioportal.org/. AACR GENIE sample data used in this study is available at https://genie.cbioportal.org/?continue. Source Data are provided with this paper.

Research involving human participants, their data, or biological material

Policy information about studies with human participants or human data. See also policy information about sex, gender (identity/presentation), and sexual orientation and race, ethnicity and racism.

Reporting on sex and gender	Tumor samples included in this study were not chosen based on sex or gender, but were instead chosen based on tissue availability. 2,639 tumors samples were from females and 2,232 from males. Sex was self-reported by the patient. The findings in this paper are applicable to both sexes. Gender information was not collected as it was not a primary aim of this study.
Reporting on race, ethnicity, or other socially relevant groupings	Race, ethnicity, and or other socially relevant groupings were not collected as it was not a primary aim of this study.
Population characteristics	Population characteristics were not collected as it was not relevant to the analysis.
Recruitment	No specific recruitment was utilized for this study. Patients included in this study were seen by MSKCC oncologists prospectively and sequenced accordingly. Patient's receiving MSK-IMPACT testing signed a clinical consent form and was enrolled on an institutional IRB-approved research protocol (MSKCC; NCT01775072). This may have potentially led to biases based on the population of patients at MSK, specifically as it relates to a cancer center.
Ethics oversight	Memorial Sloan Kettering Cancer Center Institutional Review and Privacy Board

Note that full information on the approval of the study protocol must also be provided in the manuscript.

Field-specific reporting

Please select the one below that is the best fit for your research. If you are not sure, read the appropriate sections before making your selection.

☒ Life sciences ☐ Behavioural & social sciences ☐ Ecological, evolutionary & environmental sciences

For a reference copy of the document with all sections, see nature.com/documents/nr-reporting-summary-flat.pdf

Life sciences study design

All studies must disclose on these points even when the disclosure is negative.

Sample size	Tumor genomic data from 4,871 cancer patients with requests for MSK-IMPACT testing was used. A sample size determination was not performed as all cytology samples tested were included in the study. Sample size was deemed sufficient given the large cohort of the study sample.
Data exclusions	No samples were excluded.
Replication	Each patient sample was tested once and no replication was performed for clinical testing. No replication was required as a validated clinical assay (MSK-IMPACT) was used. as the results are used for clinical treatment with ongoing validation and quality checks the results are deemed sufficiently accurate to not require replication.
Randomization	Randomization was not relevant to the study as no intervention was part of the analysis and all patient's/samples. were tested on the same assay analyzed (MSK-IMPACT).
Blinding	Analysis was performed unblinded as cytology sample data was compared to corresponding non-cytology samples. All samples included in this study were deidentified before analysis.

Reporting for specific materials, systems and methods

We require information from authors about some types of materials, experimental systems and methods used in many studies. Here, indicate whether each material, system or method listed is relevant to your study. If you are not sure if a list item applies to your research, read the appropriate section before selecting a response.

Materials & experimental systems

n/a	Involved in the study
☒	☐ Antibodies
☒	☐ Eukaryotic cell lines
☒	☐ Palaeontology and archaeology
☒	☐ Animals and other organisms
☐	☒ Clinical data
☒	☐ Dual use research of concern
☒	☐ Plants

Methods

n/a	Involved in the study
☒	☐ ChIP-seq
☒	☐ Flow cytometry
☒	☐ MRI-based neuroimaging

Clinical data

Policy information about clinical studies

All manuscripts should comply with the ICMJE guidelines for publication of clinical research and a completed CONSORT checklist must be included with all submissions.

Clinical trial registration	NCT01775072
Study protocol	https://clinicaltrials.gov/study/NCT01775072
Data collection	Tissue samples were collected from patients who were recruited into the study and MSK-IMPACT were performed where applicable.
Outcomes	Genomic profiling results were reported back to the ordering clinician and the patient through clinical reports. This study is retrospective with the only endpoint being the result of MSK-IMPACT clinical testing.

Plants

Seed stocks	No seeds stocks were used for this study
Novel plant genotypes	This is not relevant to our study
Authentication	This is not relevant to our study
